# Thrombogenicity of flow diverters in an ex vivo shunt model: effect of phosphorylcholine surface modification

**DOI:** 10.1136/neurintsurg-2016-012612

**Published:** 2016-10-31

**Authors:** Matthew W Hagen, Gaurav Girdhar, John Wainwright, Monica T Hinds

**Affiliations:** 1 Department of Biomedical Engineering, Oregon Health & Science University, Portland, Oregon, USA; 2 Neurovascular R&D, Medtronic, Irvine, California, USA

**Keywords:** Aneurysm, Flow Diverter, Platelets, Stenosis, Technology

## Abstract

**Background:**

Flow diverters offer a promising treatment for cerebral aneurysms. However, they have associated thromboembolic risks, mandating chronic dual antiplatelet therapy (DAPT). Shield Technology is a phosphorylcholine surface modification of the Pipeline Embolization Device (PED) flow diverter, which has shown significant reductions in material thrombogenicity in vitro.

**Objective:**

To compare the thrombogenicity of PED, PED with Shield Technology (PED+Shield), and the Flow-Redirection Endoluminal Device (FRED)—with and without single antiplatelet therapy and DAPT—under physiological flow.

**Methods:**

An established non-human primate ex vivo arteriovenous shunt model of stent thrombosis was used. PED, PED+Shield, and FRED were tested without antiplatelet therapy, with acetylsalicylic acid (ASA) monotherapy, and with DAPT. Radiolabeled platelet deposition was quantified over 1 hour for each device and total fibrin deposition was also quantified.

**Results:**

Cumulative statistical analysis showed significantly lower platelet deposition on PED compared with FRED. The same statistical model showed significant decreases in platelet deposition when ASA, clopidogrel, or Shield Technology was used. Direct comparisons of device performances within antiplatelet conditions showed consistent significant decreases in platelet accumulation on PED+Shield relative to FRED. PED+Shield showed significant reductions in platelet deposition compared with unmodified PED without antiplatelet therapy and with DAPT. PED accumulated minimal fibrin with and without Shield Technology.

**Conclusions:**

In this preclinical model, we have shown that the Shield Technology phosphorylcholine modification reduces the platelet-specific thrombogenicity of a flow diverter under physiologically relevant flow with and without DAPT. We have further identified increased fibrin-driven thrombogenicity associated with FRED relative to PED.

## Introduction

Coiling and stent-assisted coiling have been the ‘gold standard’ for the endovascular treatment of cerebral aneurysms.^1^ Flow diversion devices are a recent and significant shift in the treatment of these vascular anomalies.^2^ ^3^ Unlike intrasaccular devices, flow diverters consist of a highly porous metal stent deployed in the parent artery covering the aneurysm neck in order to divert blood flow away from the aneurysm—thereby driving the gradual thrombosis and healing of the aneurysm sac over time. Owing to the approximately 30% metal coverage of the vessel wall, these devices also provide a scaffold for endothelialization across the aneurysm neck.^2^ The only flow diversion device with Food and Drug Administration approval is the Pipeline Embolization Device (PED). However, other devices including the Flow-Redirection Endoluminal Device (FRED) are available in Europe and in US clinical trials. Numerous clinical studies and case reports have demonstrated the effectiveness of PED^4–6^ and FRED,^7^ ^8^ respectively, in aneurysm treatment. In this study, we compared a dual-layered flow diverter (FRED) with single-layer diverters (PED and PED with Shield Technology) to understand the contribution of flow-mediated thrombogenicity.

Thromboembolic risk is associated with the use of coiling,^9^ stent assisted coiling,^10^ and flow diverters.^11^ ^12^ The use of endoluminal devices, including flow diverters, requires perioperative systemic heparinization and dual antiplatelet therapy (DAPT: acetylsalicylic acid (ASA) with clopidogrel) before, and for at least 3 months after device deployment, with some patients requiring at least one antiplatelet therapy for life. In addition to generally increasing the risk of bleeding, this level of anticoagulation limits the effective acute use of flow diverters for ruptured aneurysms.^2^ Another limitation is that clopidogrel metabolism is dependent on the activity of the cytochrome P450 CYP2C19. Common polymorphisms affecting CYP2C19 activity result in widely variable effects of clopidogrel on platelet activity across populations, potentially reducing antiplatelet effect in some cases.^13^

To improve hemocompatibility there is an interest in the development of thromboresistant flow diverters. Phosphorylcholine is a major component of the erythrocyte outer cell membrane and has demonstrated efficacy in resisting platelet adhesion^14^ and intimal hyperplasia^15^ on arterial grafts. Medtronic, Inc has developed a new phosphorylcholine surface modification known as Shield Technology that is 3 nm thick and is covalently bound to the PED braid. In vitro studies of PED with Shield Technology have shown dramatic reductions in material thrombogenicity as indicated by reduced thrombin generation under static conditions.^16^ However, the thrombogenicity of Shield Technology under physiological flow conditions in the absence of systemic anticoagulants and in the presence of antiplatelet therapies remains unknown.

This study uses a well-established non-human primate ex vivo arteriovenous shunt model^17^ to quantify the relative thrombogenicity of three flow diversion devices: PED, PED with Shield Technology (PED+Shield), and FRED. Devices were tested without antiplatelet therapy, with ASA alone, and with DAPT (ASA with clopidogrel). Thrombogenicity was assessed by quantification of radiolabeled platelets over time and total fibrin deposition on devices.

## Materials and methods

### Devices

All devices tested were sterilized final products. The following flow diversion devices were tested: (a) Pipeline Flex Embolization Device (PED, N=9, 5 mm × 35 mm, Medtronic); (b) Pipeline Flex Embolization Device with Shield Technology (PED+Shield, N=9, 5 mm×35 mm, Medtronic); (c) FRED (N=8, 5 mm×36 mm, MicroVention). Devices were deployed in silastic medical grade tubing (3.98 mm internal diameter, Technical Products, Inc).

### Arteriovenous shunt placement

Survival studies were conducted using a single male juvenile baboon (*Papio anubis*). Experiments were approved by our institutional Animal Care and Use Committee according to guidelines of the National Institutes of Health “Guide for the Care and Use of Laboratory Animals” prepared by the Committee on Care and Use of Laboratory Animals of the Institute of Laboratory Animal Resources, National Research Council (International Standard Book, number 0-309-05377-3, 1996). Animal care has been described in detail elsewhere.^18^

A silicone shunt was surgically implanted between the femoral artery and femoral vein. For this procedure, anesthesia was inducted by ketamine (10–20 mg/kg intramuscularly) and Telazol (3–5 mg/kg intramuscularly) and maintained with 1–3% isoflurane delivered at 1–2 L/min in oxygen. Over the course of this study, each leg was independently shunted, with at least 5 days between surgery and shunt studies.

### Arteriovenous shunt studies

Autologous platelets were radiolabeled with ^111^In and re-infused weekly, as described previously.^18^ Homologous fibrinogen was radiolabeled with ^125^I and injected daily, before each study.^19^ A complete blood count was performed daily to track the animal's platelet count and calculate platelet deposition from the ^111^In signal. For the studies, the femoral arteriovenous shunt loop was extended with silicone tubing (Technical Products, Inc, 760 mm long, 3.18 mm internal diameter, 6.35 mm outer diameter (OD)). Each deployed flow diverter was connected to the shunt loop and centered over a gamma scintillation camera (model 400 T Maxi-Camera; GE) which quantified ^111^In deposition in 3-min frames over each 60-min study. The rate of blood flow through the shunt loop was monitored with an ultrasonic flow probe (Transonic Systems, Inc) proximal to the test device and maintained at 0.1 L/min (shear rate of 265 s^−1^) using a clamp distal to the device (see online supplementary figure S1).

10.1136/neurintsurg-2016-012612.supp2 Supplementary data

**Figure 1:**
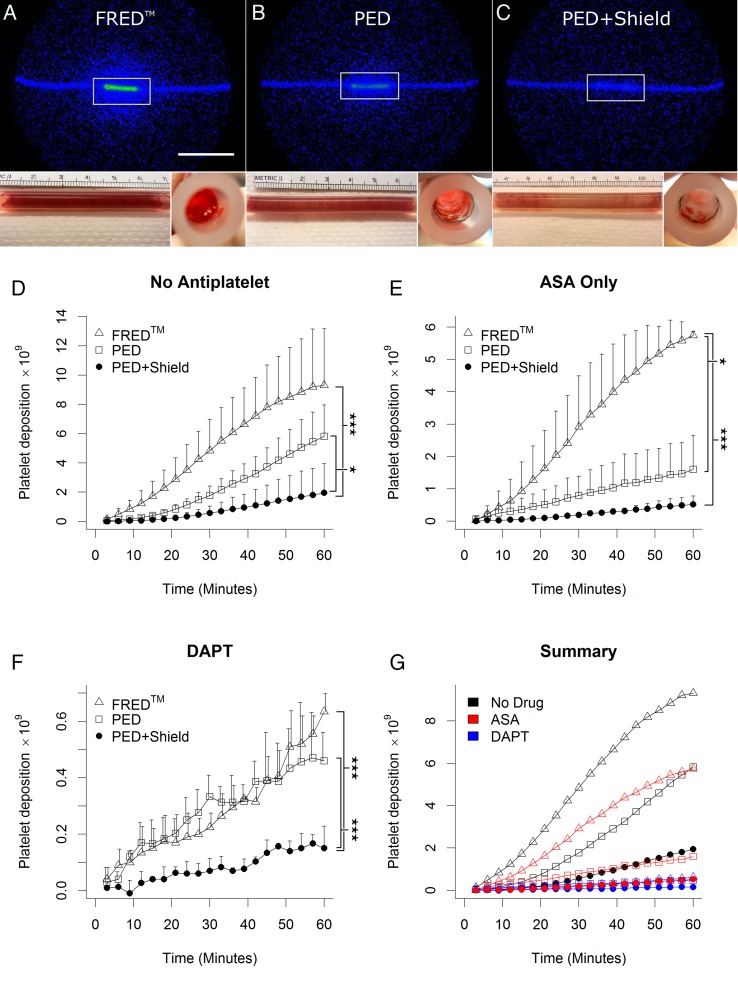
Representative scintillography and photographs with quantification of platelet deposition. (A–C) Representative scintillography and photographs from no-antiplatelet trials. (A) Flow-Redirection Endoluminal Device (FRED); (B) Pipeline Embolization Device (PED); (C) PED+Shield. Main panels: gamma camera output at 60 min. Scale bar: 10 cm. White boxes show region used for signal quantification. Insets: photographs of devices inside silicone tubing at the conclusion of arteriovenous shunt studies. Insets: (left) longitudinal and (right) distal end-on photographs of devices at the end of shunt studies. (D–G) Summary of platelet deposition data from all trials. In panels D–F, symbols represent means and error bars represent +SD. (D) In the absence of antiplatelet therapy, PED+Shield devices showed a significant decrease in platelet deposition relative to PED (p=0.016*) and FRED (p<0.001***). (E) After acetylsalicylic acid (ASA) monotherapy, FRED devices experienced significantly more platelet deposition than either PED (p=0.022*) or PED+Shield (p<0.001***). (F) Under dual antiplatelet therapy (DAPT) PED+Shield devices experienced significantly less platelet deposition than PED (p<0.001***) or FRED (p***<0.001), which were statistically indistinguishable (p=1.0). (G) Summary of all three antiplatelet therapy conditions. Error bars have been removed for clarity.

Real-time platelet deposition was calculated within a 10 cm box centered on the device (figure 1A–C) and normalized to the animal's platelet count. In the one case of shunt occlusion before the end of experiment, the platelet deposition data at the time of occlusion were duplicated for the unmeasurable time slot. Cumulative fibrin deposition was assayed by measuring ^125^I activity in a 1480 Wizard Gamma Counter (PerkinElmer) following the complete extinction of ^111^In signal from platelets. Devices were also photographed proximally, distally, and longitudinally at the conclusion of shunt studies (figure 1A–C; see online supplementary figure S3). With the exception of FRED with DAPT (which was run in duplicate) all unique drug and device combinations were run in triplicate.

### Antiplatelet drug dosing and aggregometry response

Three antiplatelet conditions were tested in this study: DAPT (ASA with clopidogrel), ASA alone, and no antiplatelet. For ASA and DAPT trials, ASA (Bayer Healthcare, LLC) was given orally at 10 mg/kg at least 4 hours before the experiment start time. Clopidogrel (Torrent Pharmaceuticals, Ltd) was given orally at 2 mg/kg twice before each DAPT study: the afternoon before and on the morning of the experiment at least 3 hours before procedures began. Prior studies performed in this model have proved this antiplatelet regimen to be effective over the duration studied here.^20^ More than 1 month passed between ASA or DAPT trials and the resumption of antiplatelet-free trials, while at least 7 days passed between DAPT and ASA monotherapy trials.^20^ Light transmission platelet aggregometry (Chronolog Corporation) was used to confirm the platelet response to each drug condition (see online supplementary methods).

### Statistical methods

Statistical calculations were performed using R (R Core Team). Platelet deposition data were log-transformed before analysis to approximate the normal distribution for parametric testing. In order to incorporate all of the time series data, platelet deposition analyses were performed using repeated-measures analysis of variance (RMA). A cumulative statistical model took PED without antiplatelet therapy as its baseline and calculated coefficients defining the effects of (1) FRED, (2) ASA and/or (3) clopidogrel, and (4) modification with Shield Technology. Clopidogrel effects were determined using this statistical model and were not separately measured. Subset RMAs were generated for each antiplatelet group (no antiplatelet, ASA, DAPT). All p values reported for platelet deposition were Bonferroni-corrected to compensate for using each datum in two models. Direct comparisons between conditions within subset analyses were performed using linear combination t-tests with an additional Bonferroni correction for multiple comparisons. Platelet aggregometry data were analyzed using a two-way RMA (on drug presence vs absence and aggregation time). Fibrin data were analyzed within antiplatelet drug groups using a one-way analysis of variance on device. Direct comparisons of fibrin data were performed using the Dunnett test, taking unmodified PED as a reference. All reported values are mean± SD at 60 min. Significance was defined as p≤0.05.

## Results

### Platelet deposition onto FRED devices is increased in the absence of clopidogrel

Without antiplatelet therapy (see online supplementary results for antiplatelet efficacy confirmation), subset analysis shows an increase in platelet deposition on FRED relative to PED, falling just short of statistical significance (table 1, figure 1D; p=0.084; PED: 5.8±2.1×10^9^ platelets; FRED: 9.31±3.9×10^9^ platelets). Notably, the single total device occlusion in this study occurred in a FRED without antiplatelet trial at 57 min. With ASA monotherapy there was a significant increase in platelet deposition seen on FRED (figure 1E; p=0.022; PED: 1.6±1.1×10^9^ platelets; FRED: 5.74±0.13×10^9^ platelets). However, no platelet deposition difference was seen between PED and FRED under DAPT (figure 1F; p=1.0; PED: 0.46±0.1×10^9^ platelets; FRED: 0.64±0.06×10^9^ platelets). Thus, while statistical significance was achieved only in ASA trials, FRED showed a trend towards greater platelet accumulation in the absence of clopidogrel.

**Table 1:** Summary of platelet deposition statistical analyses. The comprehensive analysis calculates specific effect sizes for FRED, Shield Technology, ASA, and Clopidogrel using unmodified PED without antiplatelet therapy as a reference. Subset analyses are direct comparisons between devices (FRED, PED, PED+Sheild) within each antiplatelet therapy (no antiplatelet, ASA alone, DAPT)

Comparison	Effect (platelets×10^9^)	Test statistic	p Value
Comprehensive analysis vs PED alone			
FRED	+2.01	F=34.99	1.4×10^−5^***
Shield Technology	−2.44	F=17.32	8.1×10^−4^***
ASA	−1.87	F=23.95	1.9×10^−5^***
Clopidogrel	−2.41	F=33.62	4.8×10^−4^***
Subset: no antiplatelet			
FRED vs PED	2.32	t=2.20	0.084
FRED vs PED+Shield	6.73	t=4.99	1.8×10^−6^***
PED vs PED+Shield	2.9	t=2.79	0.016*
Subset: ASA alone			
FRED vs PED	2.92	t=2.68	0.022*
FRED vs PED+Shield	7.04	t=4.88	3.2×10^−6^***
PED vs PED+Shield	2.41	t=2.20	0.084
Subset: DAPT			
FRED vs PED	−1.01	t=−0.08	1.00
FRED vs PED+Shield	1.46	t=4.59	1.4×10^−5^***
PED vs PED+Shield	2.07	t=4.89	3.0×10^−6^***

*denotes p<0.05; ***denotes p<0.001.

ASA, acetylsalicylic acid; DAPT, dual antiplatelet therapy; FRED, Flow-Redirection Endoluminal Device; PED, Pipeline Embolization Device.

### Shield Technology decreased platelet deposition across drug conditions

Without antiplatelet therapy, subset analysis associates Shield Technology with a significant reduction in platelets deposited (figure 1D; p=0.016; PED: 5.8±2.1×10^9^ platelets; PED+Shield: 2.0±2.0×10^9^ platelets). Under ASA monotherapy, the difference between PED with and without Shield Technology fell just short of significance (figure 1E; p=0.084; PED: 1.6±1.1×10^9^ platelets; PED+Shield: 0.52±0.26×10^9^ platelets). However, under DAPT a significant reduction was attributable to Shield Technology (figure 1F; p<0.001; PED: 0.46±0.1×10^9^ platelets; PED+Shield: 0.15±0.08×10^9^ platelets). The Shield Technology surface modification of PED provides a consistent reduction in platelet deposition across drug conditions. However, the magnitude of this effect is variable, as indicated by our failure to achieve statistical significance with ASA monotherapy.

### Cumulative analysis reveals significant thromboresistance by Shield Technology

**Figure 2:**
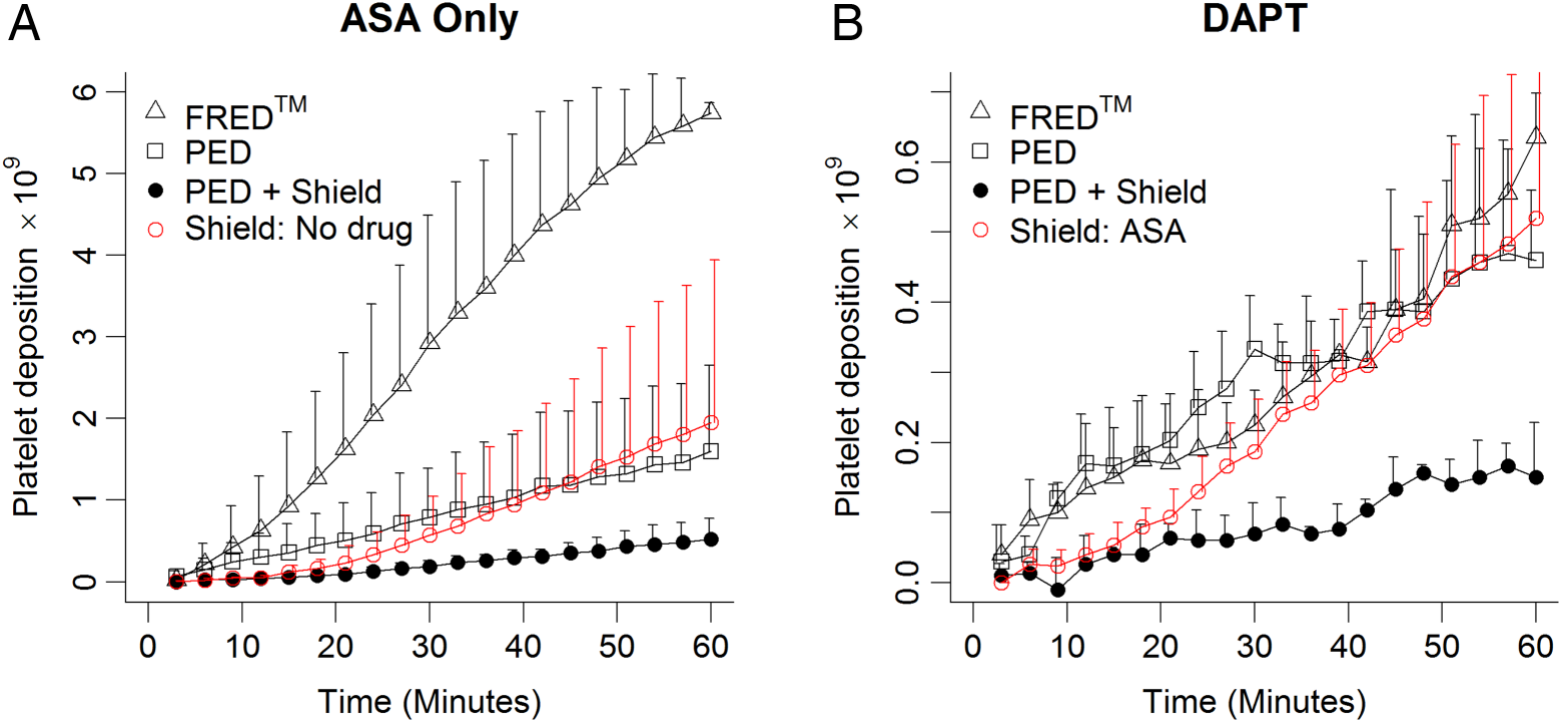
Specific effectiveness of Shield Technology. Summary of Shield Technology performance with reduced antiplatelet therapy compared with other device classes. (A) Platelet deposition onto Shield devices without any antiplatelet therapy (red) is compared with all devices under acetylsalicylic acid (ASA) monotherapy (black). (B) Shield with ASA monotherapy (red) is compared with all devices under dual antiplatelet therapy (DAPT; black). Shield Technology, ASA, and clopidogrel were each associated with significant antiplatelet effects in our cumulative analysis of all platelet deposition trials. FRED, Flow-Redirection Endoluminal Device; PED, Pipeline Embolization Device.

All platelet deposition data were analyzed together using bare PED without antiplatelet therapy, which had 5.8±2.1×10^9^ platelets attached at 60 min as a reference. Specific effect sizes on platelet attachment were calculated for ASA, clopidogrel, FRED, and Shield Technology. ASA treatment was associated with a reduction of 1.87±1.2×10^9^ platelets (p<0.001) while clopidogrel treatment and presence of Shield Technology on PED were associated with reductions of 2.41±1.3×10^9^ and 2.44±1.2×10^9^ platelets, respectively (both: p<0.001). In contrast, FRED was associated with an increase of 2.01±1.25×10^9^ platelets (p<0.001). Thus, the use of ASA, clopidogrel, and Shield Technology individually each showed statistically significant thromboresistant effects compared with PED alone (figure 2).

### FRED accrues significantly more fibrin than PED

**Figure 3:**
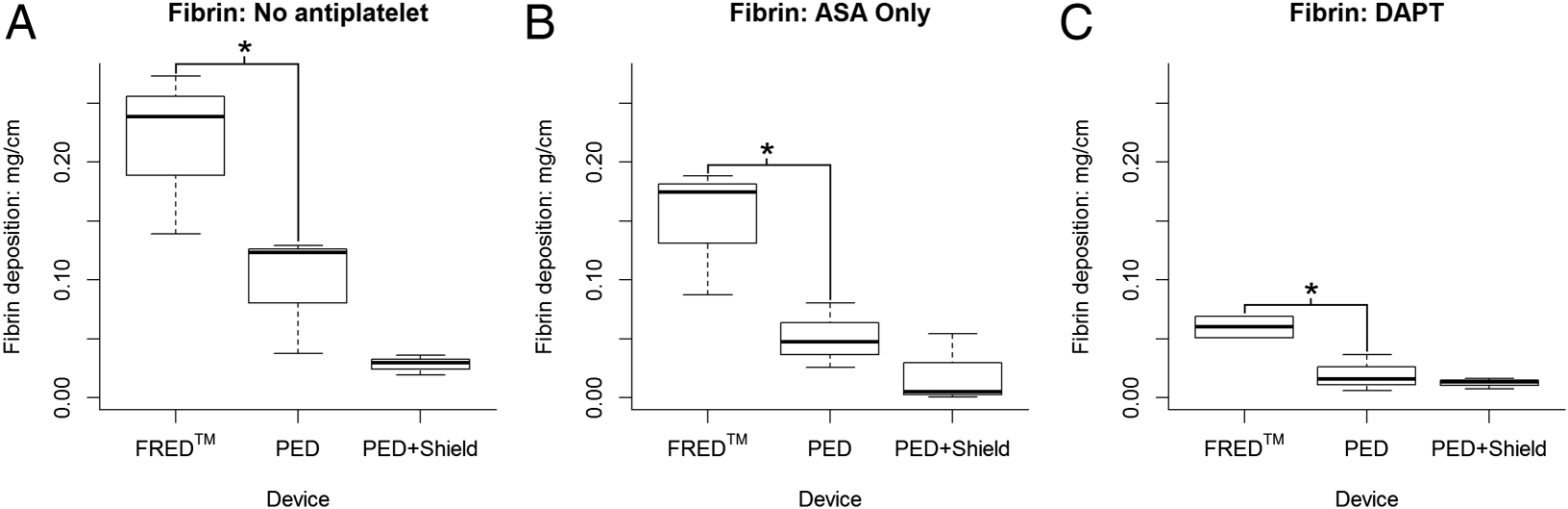
Fibrin deposition. Across all drug conditions tested, Flow-Redirection Endoluminal Device (FRED) devices showed significantly greater fibrin accumulation than the Pipeline Embolization Device (PED). Shield Technology did not have a significant impact on fibrin deposition relative to unmodified PED. (A) In the absence of antiplatelet therapy, FRED showed significantly greater fibrin deposition than PED (p=0.045*). (B) Under acetylsalicylic acid (ASA) monotherapy, FRED showed significantly greater fibrin accumulation than PED (p=0.038*). (C) Likewise, under dual antiplatelet therapy (DAPT), FRED showed significantly greater fibrin accumulation than PED (p=0.023*).

After the platelet deposition studies, total fibrin deposition on each device was quantified. FRED use was associated with an increase in fibrin deposition across all antiplatelet conditions. Without antiplatelet drugs, the amount of fibrin on FRED was more than double that seen on PED (figure 3A; p=0.045; PED: 0.097±0.051 mg/cm fibrin, FRED: 2.17±0.069 mg/cm fibrin). Under ASA monotherapy a similarly significant difference was seen between FRED and PED (figure 3B; p=0.038; PED: 0.051±0.027 mg/cm fibrin, FRED: 0.15±0.055 mg/cm fibrin), with similar results seen under DAPT (figure 3C; p=0.023; PED: 0.020±0.016 mg/cm fibrin, FRED: 0.06±0.013 mg/cm fibrin). In summary, fibrin accumulation is significantly and consistently higher on FRED than PED.

Shield Technology was associated with a small and statistically insignificant fibrin decrease relative to bare PED without antiplatelet therapy (figure 3A; p=0.240; PED: 0.097±0.051 mg/cm fibrin, PED+Shield: 0.028±0.008 mg/cm fibrin). Under ASA monotherapy and DAPT, Shield Technology use was likewise associated with small and statistically insignificant decreases in total fibrin (figure 3B, C; ASA: p=0.553; PED: 0.051±0.027 mg/cm fibrin, PED+Shield: 0.020±0.030 mg/cm fibrin; DAPT: p=0.700; PED: 0.020±0.016 mg/cm fibrin, PED+Shield: 0.013±0.005 mg/cm fibrin). Moderate fibrin decreases were seen with Shield Technology use; however, none were statistically significant.

## Discussion

### The baboon arteriovenous shunt model of stent thrombosis

This study uses a well-established baboon ex vivo arteriovenous shunt model of stent thrombosis. Previous in vitro work has identified the effects of Shield Technology on material thrombogenicity,^16^ and understanding this technology's performance in the complex environment of flowing blood is a necessary step towards a better understanding of its ability to resist thrombosis in a clinical setting. Our ex vivo minimally invasive technique can safely be used without systemic anticoagulants.^18^ Additionally, the ex vivo shunt studies allow for consistent device deployment, identical flow conditions, and are performed in the same animal for 1:1 comparisons. Furthermore, this model, which measures activated platelet and fibrin adhesion on devices as an indication of thrombogenicity, was the basis of much of the preclinical work which established DAPT as the drug regimen used for patients receiving an arterial graft today,^20^ allowing direct comparisons with this study.

### FRED devices accrue platelets and fibrin at a higher rate than PED with Shield Technology

Fibrin accumulation was found to be greater on FRED than unmodified PED devices across antiplatelet conditions, and our cumulative analysis of platelet deposition data found that FRED use was associated with a similarly significant increase in platelet attachment. Notably, however, in subset analyses the difference between FRED and unmodified PED reached statistical significance only under ASA monotherapy, and the devices performed equivalently under DAPT (table 1).

The PED is a single-layer cobalt–chromium braid, providing approximately 30% surface coverage, with platinum wires incorporated for radio-opacity. In contrast, FRED is a dual-layer nitinol device consisting of a low-porosity inner braid attached to a high-porosity outer braid with a double helix of radiopaque tantalum wires.^12^ The material thrombogenicities of PED and FRED were previously shown to be equivalent in a static measurement of thrombin generation,^16^ therefore the differences in thrombogenicity observed here are probably flow-related. This may be attributable to the structure of FRED, as the dual-layered design may lead to localized disturbed flow conditions, which could entrap activated platelets causing a nidus for thrombus formation.

### Shield Technology has a significant platelet-specific thromboresistant effect

In this study, the effective thromboresistance of Shield Technology, a phosphorylcholine surface modification mimicking the erythrocyte outer membrane, was demonstrated. PED with Shield Technology was shown to resist in-stent thrombosis caused by the deposition of activated platelets on the surfaces of bare Pipeline devices. ASA, clopidogrel, and Shield Technology, all individually offered a significant resistance to platelet deposition. This is evident from the comparisons shown in figure 2 for ASA monotherapy and DAPT. Specifically, platelet deposition on PED+Shield without antiplatelet therapy was similar to PED with ASA monotherapy (figure 2A), and PED+Shield with ASA alone exhibited similar platelet deposition as FRED and bare PED under DAPT (figure 2B).

### PED devices accrue minimal fibrin compared with FRED

FRED showed significantly higher fibrin deposition than PED or PED+Shield across all antiplatelet conditions (figure 3). The modification of PED with Shield Technology was associated with a consistent, although never statistically significant, fibrin decrease relative to bare PED across all antiplatelet conditions. Harker *et al*,^20^ published a previous study using this baboon model which established ASA with clopidogrel as the standard DAPT following coronary stenting. The lowest level of mean fibrin accumulation they observed was 0.08 mg/cm on a bare stainless steel stent after 10 mg/kg ASA and 20 mg/kg clopidogrel pretreatment (equivalent to our ASA dosing and 10 times our clopidogrel dosing).^20^ Here, in contrast, bare PED showed mean fibrin accumulation of only 0.020 mg/cm with DAPT. Thus, while it is possible that Shield Technology confers fibrin resistance which this study failed to capture, there is little room for improvement over the current performance of PED as discernible in this model at 60 min.

### Study limitations

This study provides a direct comparison of the thrombogenicity of different flow diversion devices under physiological flow and in the presence of systemic antiplatelet therapy. However, we are aware of several limitations to our design. All of the experiments reported here were conducted in a single baboon. Owing to their high cost and associated ethical considerations, it is usual for non-human primate studies to have a small sample size. Because one of the problems with clopidogrel use clinically is the wide variability in antiplatelet efficacy associated with cytochrome P450 polymorphisms,^13^ we elected to conduct our studies in a single animal to reduce variability rather than risk the appearance of between-subjects artifacts. We used light transmission platelet aggregometry to confirm that our subject was a normal responder. The sample size of three was based on historical data from this same model in which N=3 yielded a power of 90% with α=0.05 in a demonstration of clopidogrel efficacy against platelet deposition.^20^

Our quantification of platelet and fibrin deposition from flowing blood provides a useful picture of the risks of device thrombosis; however, the model lacks the capability to robustly quantify incidents of micro-thromboembolism. Indirect detection of macro-thromboembolic events is possible by quantifying decreases in real-time platelet attachment. In this study, a single FRED with ASA trial had a decrease in platelet attachment at the 57th minute, indicating an embolic event. Future studies in this model could incorporate a distal filter which could capture thromboemboli and microemboli, enabling their quantification.

This study is further limited by its ex vivo nature and acute timescale. While our design deals with the blood flow and coagulation elements of Virchow's triad, by conducting these trials outside the native vasculature, we excluded endothelial damage, which is particularly relevant because of potential vessel wall disruption during device delivery and deployment. Additional factors missing from this ex vivo model include aneurysm presence, vessel tortuosity, and varying boundary conditions in different areas of the cerebral vasculature. Future ex vivo studies could deal with this by moving away from a straight conduit model and instead using more complex 3D models, such as those used in early studies of flow diverter fluid dynamics.^21^ Given that one goal of flow diverter use is the endothelialization of the aneurysm neck,^2^ it will also be important to assess whether Shield Technology is a substrate which encourages endothelial cell ingrowth while resisting potentially stenotic intimal hyperplasia.

## Conclusion

Using a well-established ex vivo baboon arteriovenous shunt model of stent thrombosis, we have shown that Shield Technology substantially reduces platelet deposition. We have furthermore demonstrated differences in the platelet- and fibrin-mediated thrombogenicity of PED and FRED devices, which we hypothesize are flow-related given their previously reported equivalent performances in a static study of material thrombogenicity. While the platelet-specific thromboresistance conferred by Shield Technology seen in this acute study is encouraging, the clinical use of these devices will depend on several other factors outside the scope of this model.
