# Supplementary material for: Thrombogenicity of flow diverters in an ex vivo shunt model: effect of phosphorylcholine surface modification
Source: J Neurointerv Surg. 2016 Oct 31;9(10):1006–11. doi: 10.1136/neurintsurg-2016-012612 (PMC5629931; doi:10.1136/neurintsurg-2016-012612)
Supplement: Supplementary data [file neurintsurg-2016-012612supp002.pdf]

Supplemental methods

Light transmission platelet aggregometry

Antiplatelet drug activity was confirmed using light transmission platelet aggregometry. For at least two shunt studies in each antiplatelet group (no antiplatelet, ASA alone, DAPT), baboon arterial blood was drawn from the arteriovenous shunt (9:1v/v into 3.8% sodium citrate). Platelet rich plasma (PRP) and platelet poor plasma (PPP) were isolated via consecutive centrifugations. Following a CBC on raw PRP, a working solution containing 2×10^5 platelets/µL was prepared by diluting PRP in PPP. Platelet aggregometry was performed using a Model 440 Dual Aggregometer (Chrono-Log Corporation) connected to a Model 707 chart recorder (Chrono-Log). Remaining PPP was used as an absorbance reference. Light transmission was recorded for at least 30 s prior to agonist addition. The ASA effect was assayed by adding arachidonic acid (Bio/Data Corp) to a final plasma concentration of 613 µmol/L, while clopidogrel activity was assayed with 80 µmol/L adenosine diphosphate (ADP, Sigma Chemical Co.). Light transmission was recorded for at least four minutes after agonist addition. For the purposes of quantification, percent aggregation was calculated at 90s and 180s after agonist addition. On days when no platelet response was expected based on antiplatelet use, venous blood from an untreated baboon was used as an agonist positive control.

Supplemental results

Antiplatelet therapy was effective in study subject.

Light transmission platelet aggregometry was used to confirm the inhibitory effects of ASA and clopidogrel on platelet receptors for AA and ADP, respectively. Aggregometric traces in the absence of ASA treatment showed a full response to AA (Figure S1A), while traces generated following either ASA monotherapy or DAPT yielded a total abolition of that response (Figure S1B). Quantification of percent aggregometry at 90s and 180s after AA addition revealed a significant difference between ASA groups (Figure S1C; $p=0.012$). Likewise, traces generated in the absence of clopidogrel showed a full response to

80 µM ADP (Figure S1D), while those generated following DAPT showed a total loss of the aggregative phase of the curve (Figure S1E). Quantification of percent aggregometry at 90s and 180s following ADP addition confirmed a significant difference between clopidogrel groups (Figure S1F; p=0.002). Thus the animal was a normal responder to both ASA and clopidogrel, and an appropriate experimental subject for this study.

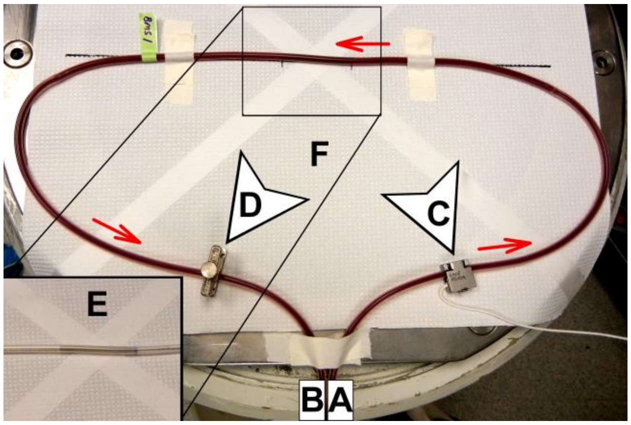

Figure S1: *Ex vivo* shunt loop setup

Representative photograph of arteriovenous *ex vivo* shunt loop during study. (A) Blood flows from the baboon femoral artery and through the shunt loop counter-clockwise (illustrated by red arrows). (B) Blood is returned to the femoral vein. (C) Blood flow is monitored using an ultrasonic flow probe proximal to the test device. (D) Blood flow is regulated using a manual clamp distal to the test device. (E inset) Test device position within shunt loop. (F) The shunt loop sits above a gamma camera which records the deposition of ^{111}In-labeled platelets on the test device.

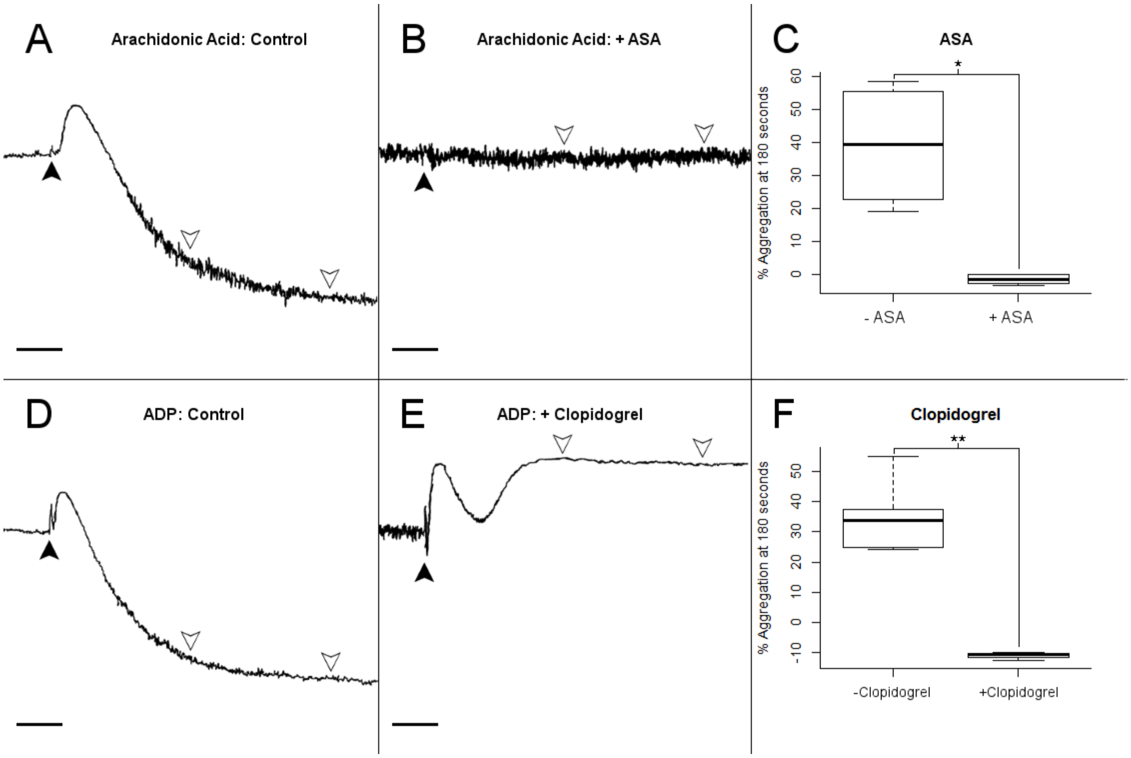

Figure S2: Platelet aggregometry

Light transmission platelet aggregometry was used to confirm the antiplatelet effects of ASA (A-C) and clopidogrel (D-F). (A) In the absence of ASA, 613 µmol/L AA induces platelet aggregation, as shown by the drop in light absorption. (B) Following ASA therapy, AA induces no response in platelets. (C) 180 seconds after agonist addition ASA has a statistically significant effect on platelet aggregation in response to AA ($p = 0.012$ *). (D) In the absence of clopidogrel, ADP induces platelet aggregation. (E) Following clopidogrel therapy, the aggregative portion of the ADP response curve is abolished. (F) Clopidogrel has a statistically significant effect on platelet aggregation in response to ADP ($p = 0.002$ **). In panels A, B, D and E, black arrows mark the time of agonist addition to PRP. Open inverted arrows mark the 90 and 180 second timepoints used for quantification. Scale bars: 30 seconds.

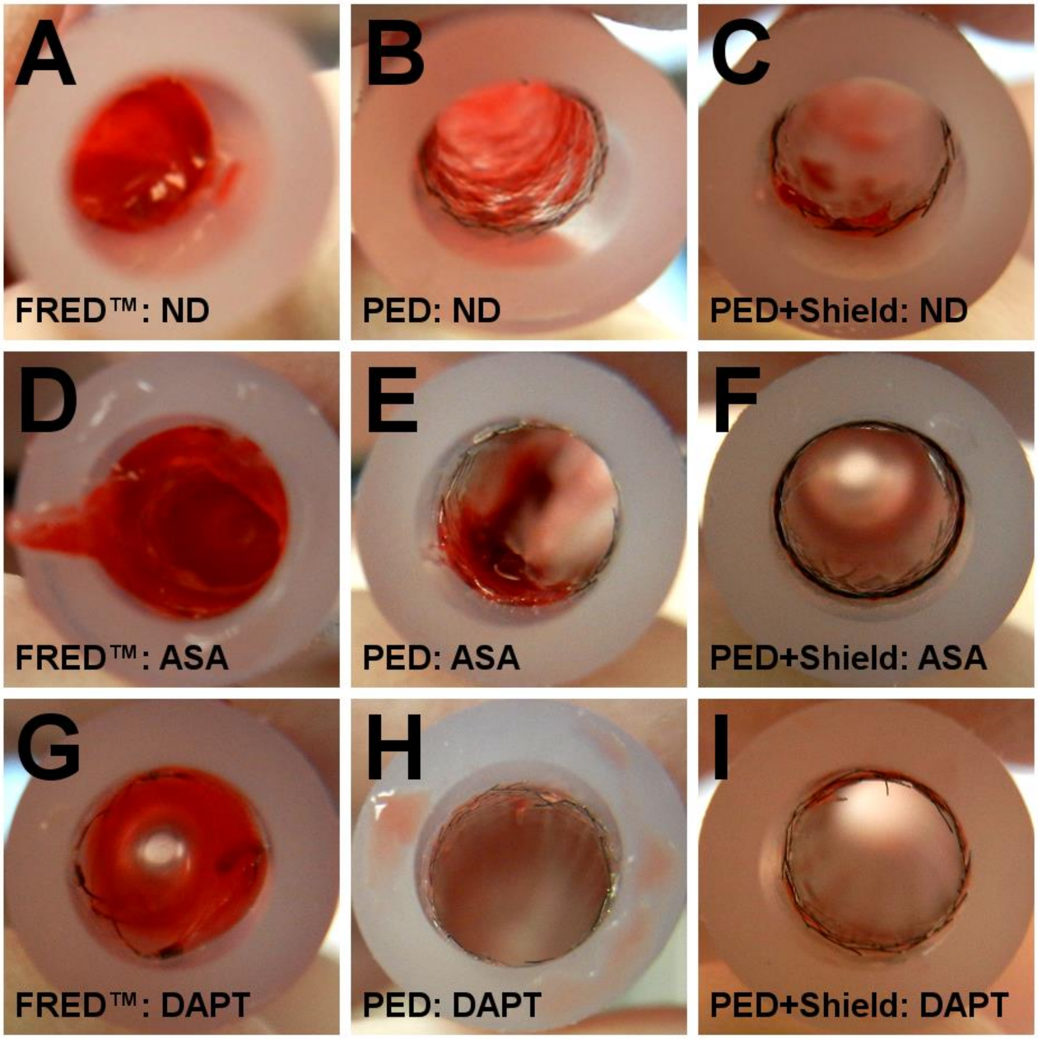

Figure S3: Device thrombus photographs

Devices were photographed at the conclusion of arteriovenous shunt studies. All images collected here are taken from the distal end looking into the device proximally. (A-C) are images of (A) FRED™, (B) PED, and (C) PED+Shield without antiplatelet treatment and are repeated form Figure 2. (D-F) show (D) FRED™, (E) PED, and (F) PED+Shield with ASA monotherapy. (G-I) show (G) FRED™, (H) PED, and (I) PED+Shield with DAPT.
